# Inhibition of Cardiac Sympathetic Afferent Reflex and Sympathetic Activity by Baroreceptor and Vagal Afferent Inputs in Chronic Heart Failure

**DOI:** 10.1371/journal.pone.0025784

**Published:** 2011-10-03

**Authors:** Xian-Bing Gan, Yang-Can Duan, Xiao-Qing Xiong, Peng Li, Bai-Ping Cui, Xing-Ya Gao, Guo-Qing Zhu

**Affiliations:** 1 Department of Physiology, Nanjing Medical University, Nanjing, China; 2 Key Laboratory of Cardiovascular Disease and Molecular Intervention, Nanjing Medical University, Nanjing, China; 3 Department of Medical Ultrasound, Affiliated Hospital of Jining Medical University, Jining, China; University of Cincinnati, United States of America

## Abstract

**Background:**

Cardiac sympathetic afferent reflex (CSAR) contributes to sympathetic activation and angiotensin II (Ang II) in paraventricular nucleus (PVN) augments the CSAR in vagotomized (VT) and baroreceptor denervated (BD) rats with chronic heart failure (CHF). This study was designed to determine whether it is true in intact (INT) rats with CHF and to determine the effects of cardiac and baroreceptor afferents on the CSAR and sympathetic activity in CHF.

**Methodology/Principal Findings:**

Sham-operated (Sham) or coronary ligation-induced CHF rats were respectively subjected to BD+VT, VT, cardiac sympathetic denervation (CSD) or INT. Under anesthesia, renal sympathetic nerve activity (RSNA) and mean arterial pressure (MAP) were recorded, and the CSAR was evaluated by the RSNA and MAP responses to epicardial application of capsaicin. Either CSAR or the responses of RSNA, MAP and CSAR to Ang II in PVN were enhanced in CHF rats treated with BD+VT, VT or INT. Treatment with VT or BD+VT potentiated the CSAR and the CSAR responses to Ang II in both Sham and CHF rats. Treatment with CSD reversed the capsaicin-induced RSNA and MAP changes and the CSAR responses to Ang II in both Sham and CHF rats, and reduced the RSNA and MAP responses to Ang II only in CHF rats.

**Conclusions:**

The CSAR and the CSAR responses to Ang II in PVN are enhanced in intact CHF rats. Baroreceptor and vagal afferent activities inhibit CSAR and the CSAR responses to Ang II in intact Sham and CHF rats.

## Introduction

Excessive sympathetic activity contributes to haemodynamic deterioration in chronic heart failure (CHF) [1], [2]. Cardiac sympathetic afferent reflex (CSAR) is known to increase sympathetic activity and blood pressure [3] and the enhanced CSAR is involved in the sympathetic over-activation in CHF [4], [5]. The CSAR can be induced by stimulation of cardiac sympathetic afferents with exogenous chemicals such as capsaicin, bradykinin, adenosine and hydrogen peroxide or endogenous chemicals released from myocardium during myocardial ischemia [3]. The enhanced CSAR in CHF attributes to the increased activity of cardiac sympathetic afferents and the potentiated central gain of this reflex [4].

Paraventricular nucleus (PVN) of hypothalamus is an important component of the central neurocircuitry of the CSAR [6], [7] and plays an important role in regulating the sympathetic and cardiovascular activity via its projections to the rostral ventrolateral medulla and intermediolateral column of the spinal cord [8]. Lots of signal molecules or enzymes in the PVN are involved in mediating or modulating the CSAR such as superoxide anions [9], NAD(P)H oxidase [10], hydrogen peroxide [11], GABA [12], c-Src [13], tumor necrosis factor α and interleukin 1β [14]. It has been found that angiotensin II (Ang II) type 1 (AT_1_) receptor expression is increased in the PVN in CHF rats [15]. Ang II in the PVN augments the CSAR and increases the sympathetic outflow and blood pressure, which is mediated by AT_1_ receptors in the PVN in CHF rats [16]. The effects of Ang II in the PVN on the CSAR and sympathetic activation are mediated by the NAD(P)H oxidase originated superoxide anions in the PVN in CHF rats and renovascular hypertensive rats [17], [18]. However, these experiments were carried out in animals with bilateral cervical vagotomy (VT) and baroreceptor denervation (BD) to minimize the confounding effects of baroreceptor and vagal afferent activities on the CSAR and sympathetic drive in these experiments. One key issue to be resolved is whether the sympatho-excitatory CSAR is still enhanced in intact CHF rats.

It is known that the sympatho-inhibitory baroreceptor reflex is diminished or desensitized whereas the sympatho-excitatory CSAR and arterial chemoreceptor reflex are enhanced in CHF [3], [5]. Augmented input from cardiac sympathetic afferents enhances the arterial chemoreceptor reflex in normal rats [19] and CHF rats [20] whereas inhibits baroreflex in normal rats [21] and CHF rats [22]. On the other hand, stimulation of cardiac vagal afferent endings evokes reflex hypotension and bradycardia [23]. However, the effects of the arterial baroreceptor and vagal afferents on the enhanced CSAR in CHF rats are unknown. Furthermore, the impacts of arterial baroreceptor and vagal inputs on the effects of Ang II in the PVN in CHF are unknown. The aims of the present study were to determine whether the CSAR was enhanced in intact CHF rats, and whether the baroreceptor and vagal afferents inhibited the enhanced CSAR and the CSAR-enhancing effects of Ang II in the PVN in CHF rats.

## Materials and Methods

Experiments were carried out on male Sprague-Dawley rats weighing between 300 and 400 g. The procedures were approved by the Experimental Animal Care and Use Committee of Nanjing Medical University (No. 20100097) and complied with the Guide for the Care and Use of Laboratory Animals (NIH publication no. 85–23, revised 1996). The rats were housed individually in standard laboratory cages with ad libitum access to standard chow and tap water under controlled temperature and a 12:12-h dark-light cycle.

### CHF model

Myocardial infarction is a common cause in driving the occurrence of ventricular dysfunction and heart failure [24]. The CHF in the present study was induced by coronary artery ligation with sterile techniques as previously reported [25]. Briefly, the rats were anesthetized with sodium pentobarbital (50 mg kg^−1^, i.p.). The rats were randomly subjected to the ligation of the left anterior descending coronary artery or sham operation. The sham-operated (Sham) rats were treated the same as the coronary ligation rats except that their coronary arteries were not ligated. The criteria for CHF is that the left ventricle end-diastolic pressure (LVEDP) is higher than 12 mm Hg and the maximum of the first differentiation of left ventricular pressure (+dP/dt _max_) is 40% lower than that of Sham rats.

### General procedures of acute experiment

Acute experiment was carried out 7 weeks after coronary ligation or sham surgery. Each rat was anaesthetized with intraperitoneal injection of urethane (800 mg kg^−1^) and α-chloralose (40 mg kg^−1^). Supplemental doses of urethane and α-chloralose were administered to maintain an adequate depth of anesthesia during experiments. The trachea was cannulated and connected with a rodent ventilator (683; Harvard Apparatus Inc., USA) for mechanical ventilation. A catheter connected with a pressure transducer was placed into the right carotid artery to measure the arterial pressure.

### Baroreceptor denervation and vagotomy

Vagotomy (VT) and arterial baroreceptor denervation (BD) were carried out as previously reported [9], [26]. The bilateral cervical vagi, carotid sinus nerves and other visible nerve fibers in carotid sinus areas were sectioned. The carotid bifurcation and common carotid arteries were stripped of adventitial tissues from 4 mm below the bifurcation to 4 mm above. The vessels were painted with 10% phenol solution to destroy remnants of nerve fibers in these areas. Baroreceptor denervation was assumed to be complete if heart rate (HR) changed less than 5 beats min^−1^ in response to intravenous injection of phenylephrine (20 µg kg^−1^).

### Cardiac sympathetic denervation

Bilateral cardiac sympathetic denervation (CSD) companied with stellate ganglionectomy was carried out as previously reported [27]. A thoracotomy was performed by cutting the sternum directly on the midline from the manubrium to just above the xiphoid process. All nerve branches running into the stellate ganglion between the first and second ribs beneath the parietal pleura were isolated and sectioned. The ganglion was excised and the T_1_–T_4_ sympathetic rami were transected.

### Renal sympathetic nerve activity recording

The renal sympathetic nerve activity (RSNA) was recorded as previously reported [28]. After the left renal nerve was distally cut, the nerve was placed onto a pair of silver recording electrodes and was soaked in warm mineral oil. The signal was amplified (×1000) using an AC/DC differential amplifier (3000; A–M Systems Inc., Sequim, WA, USA) with a band-pass filter (low frequency 30 Hz, high frequency 3 kHz). The amplified and filtered signal was integrated (10 ms time constant). The baseline noise was determined after section of the central end of the renal nerve at the end of the experiment and was subtracted from the integrated RSNA. The raw RSNA, integrated RSNA, arterial pressure, mean artery pressure (MAP) and HR were simultaneously recorded on a PowerLab data acquisition system (8SP; ADInstruments, Sydney, Australia) and stored on disk until analyzed.

### Evaluation of CSAR

The heart was exposed and the pericardium was removed through the thoracotomy. A piece of filter paper (3×3 mm) containing capsaicin (1.0 nmol in 2.0 µl) was applied to the epicardial surface of the anterior wall of the left ventricle for 1 min to induce the CSAR. Then, the epicardium was rinsed three times with 10 ml of warm normal saline (38°C). The CSAR was evaluated by the RSNA and MAP responses to epicardial application of capsaicin [26].

### PVN microinjection

The rats were placed in a stereotaxic frame (Stoelting, Chicago, IL, USA). The stereotaxic coordinates for the PVN are 1.8 mm caudal to bregma, 0.4 mm lateral to the mid-line and 7.9 mm ventral to the dorsal surface according to the stereotaxic atlas of Paxinos & Watson (2005). Microinjection into the PVN was performed with a glass micropipette (50 µm tip diameter). The microinjection volume for each side of the PVN was 50 nl and the microinjections were completed within 1 min. At the end of the experiment, the same volume of Evans Blue dye was injected into the microinjection site for histological verification. The data from the rats whose microinjection sites were outside the PVN were excluded for analysis [29].

### Protocols

Acute experiments were carried out 7 weeks after coronary ligation or sham surgery. Either Sham or CHF rats were randomly divided into four groups, which were subjected to the VT, BD+VT or CSD, or were kept intact (INT). A 60-min stabilization period was allowed before determination of the CSAR induced by epicardial application of capsaicin (1.0 nmol, n = 6 for each group) or the PVN microinjection of Ang II (0.3 nmol) followed by CSAR determination 2 min after Ang II (n = 6 for each group). For evaluation of the CSAR, the RSNA and MAP were determined before epicardial application and 20 s after epicardial application by averaging 20 s of the values.

### Drugs

Capsaicin and Ang II were purchased from Sigma.

### Statistics

The values were expressed as the means ± S.E.M. Comparisons between two observations in the same animal were assessed by Student's paired *t* test. One-way or two-way ANOVA followed by the Newman-Keuls test for post hoc analysis was used when multiple comparisons were made. Statistical significance was taken at a value of *P*<0.05.

## Results

### Anatomical and hemodynamic data

The mean infarct area was 34.0 % of the left ventricle in CHF rats but no obvious infarct was found in Sham rats. The heart weight and the heart-to-body weight ratio were increased in CHF rats, suggesting myocardial hypertrophy in the non-infarcted region of the myocardium. The systolic arterial pressure, left ventricle peak systolic pressure, developed pressure and +dP/dt_max_ deceased, but LVEDP increased in CHF rats (Table 1).

**Table 1:** Anatomic and hemodynamic data in Sham and CHF rats.

	Sham	CHF
n	48	48
Body weight (g)	358.2±6.7	351.7±6.9
Heart weight (g)	1.3±0.1	1.5±0.1 *
Heart weight/body weight (g/kg)	3.5±0.2	4.3±0.2 *
Infarct size (% LV area)	0	34.0±2.3 *
Systolic pressure (mm Hg)	127.0±4.6	111.8±4.3 *
Diastolic pressure (mm Hg)	75.5±2.9	76.1±2.8
Mean arterial pressure (MAP, mm Hg)	94.6±3.9	89.4±3.7
Heart rate (HR, beats/min)	376.9±8.1	383.3±7.2
LV systolic pressure (LVSP, mm Hg)	134.5±4.0	114.6±4.9 *
LV end-diastolic pressure (LVEDP, mm Hg)	0.3±0.9	15.0±0.9 *
+LV dP/dt_max_ (mm Hg/sec)	3510.5±106.7	1930.8±89.3 *

LV, left ventricle; +LV dP/dt_max_, maximum of the first differentiation of left ventricular pressure. Values are mean ± SE.

*P<0.05 compared with Sham rats.

### MAP and HR

No significant difference in MAP and HR was found 1 hour after treatment with INT, VT, BD+VT or CSD between Sham rats and CHF rats. However, the CSD treatment decreased the HR in CHF rats but not in Sham rats (Table 2).

**Table 2:** The MAP and HR in Sham and CHF rats 1 hour after treatment with INT, VT, BD+VT or CSD.

	Sham	CHF
MAP, mm Hg		
INT	94.2±3.8	89.8±6.3
VT	95.7±3.9	94.3±4.4
BD+VT	99.3±5.4	95.5±7.4
CSD	93.2±4.8	89.5±4.8
HR, beats/min		
INT	376.3±9.1	386.3±10.9
VT	391.3±12.3	392.3±10.2
BD+VT	390.1±11.4	396.2±12.0
CSD	368.7±9.3	354.8±10.5 *

Values are mean ± SE. n = 12 for each group.

*P<0.05 compared with INT.

### Baseline CSAR

**Figure 1:**
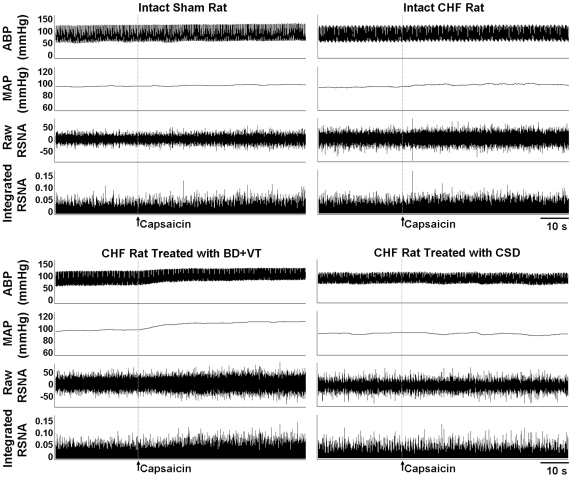
Representative tracings showing the CSAR induced by epicardial application of capsaicin in intact Sham rat, intact CHF rat, BD+VT-treated CHF rat and CSD-treated CHF rat. BD+VT, baroreceptor denervation plus vagotomy; CSD, cardiac sympathetic denervation.

**Figure 2:**
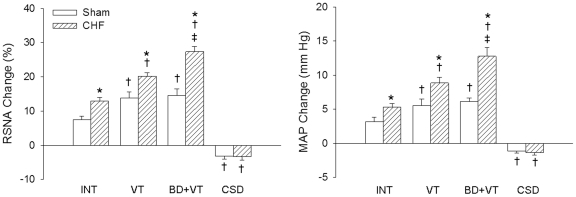
The CSAR induced by epicardial application of capsaicin in Sham and CHF rats with INT, VT, BD+VT or CSD (n  =  6 for each group). Values are mean±SE. *P<0.05 compared with Sham rats; †P<0.05 compared with INT; ‡P<0.05 compared with VT. INT, intact; BD, baroreceptor denervation; VT, vagotomy; CSD, cardiac sympathetic denervation.

Representative recordings in Fig. 1 showed the baseline CSAR change (RSNA and MAP responses to epicardial application of capsaicin) in Sham rats and CHF rats. Capsaicin increased the RSNA and MAP in INT, VT or BD+VT rats, but decreased the RSNA and MAP in CSD rats. In INT-, VT- or BD+VT-treated rats, the excitatory responses of capsaicin were greater in CHF rats than Sham rats. However, in CSD-treated rats, the inhibitory responses of capsaicin were not significantly greater in CHF rats than Sham rats. In either Sham or CHF rats, the RSNA and MAP responses to capsaicin were greater in VT-treated rats than INT rats. In CHF rats, the responses were greater in BD+VT-treated rats than either INT rats or VT rats (Fig. 2).

### RSNA and MAP responses to Ang II

**Figure 3:**
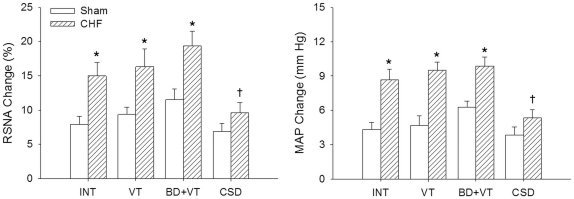
Effects of the paraventricular nucleus (PVN) microinjection of angiotensin II on the RSNA and MAP in Sham and CHF rats with INT, VT, BD+VT, or CSD (n  =  6 for each group). Values are mean±SE. *P<0.05 compared with Sham rats; †P<0.05 compared with INT. INT, intact; BD, baroreceptor denervation; VT, vagotomy; CSD, cardiac sympathetic denervation.

In INT, VT or BD+VT-treated rats, the PVN microinjection of Ang II caused greater increases in the RSNA and MAP in CHF rats than that in Sham rats. Compared with intact Sham or intact CHF rats, VT or BD+VT only caused a tendency in enhancing the effects of Ang II on the RSNA and MAP (P>0.05), while CSD attenuated Ang II-induced increases in RSNA and MAP in CHF rats but not in Sham rats (Fig. 3).

### CSAR responses to Ang II

**Figure 4:**
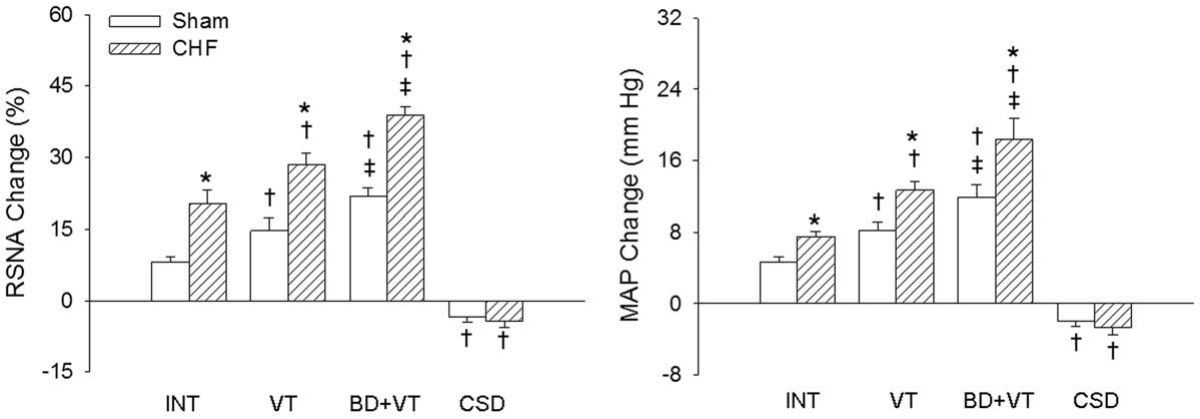
Effects of the paraventricular nucleus (PVN) microinjection of angiotensin II on the CSAR induced by epicardial application of capsaicin in Sham and CHF rats with INT, VT, BD+VT, or CSD (n  =  6 for each group). Values are mean±SE. *P<0.05 compared with Sham rats; †P<0.05 compared with INT. ‡P<0.05 compared with VT. INT, intact; BD, baroreceptor denervation; VT, vagotomy; CSD, cardiac sympathetic denervation.

The PVN microinjection of Ang II significantly augmented the RSNA and MAP responses to epicardial application of capsaicin in Sham rats treated with BD+VT and in CHF rats treated with INT, VT or BD+VT. The enhanced RSNA and MAP responses to capsaicin after Ang II were significantly greater in CHF rats than Sham rats (Table 3). The effects of Ang II in the PVN on the RSNA and MAP responses to capsaicin were augmented by VT, and further augmented by BD+VT. Ang II had no significant effects on the inhibitory RSNA and MAP responses to capsaicin in either Sham or CHF rats treated with CSD (Fig. 4).

**Table 3:** Effects of PVN microinjection of saline or Ang II on the capsaicin-induced CSAR in Sham and CHF rats.

Groups	RSNA change, %		MAP change, mmHg	
	Saline	Ang II	Saline	Ang II
Sham				
INT	7.4±1.0	8.1±1.1	3.1±0.6	4.6±0.5
VT	13.7±1.9†	14.7±2.6†	5.5±0.9†	8.1±1.0†
BD+VT	14.6±1.8†	21.8±1.8* † ‡	6.1±0.6†	11.9±1.4* † ‡
CSD	−3.2±0.8†	−3.4±1.2†	−1.2±0.3†	−2.0±0.5†
CHF				
INT	12.9±1.0#	20.4±2.7*#	5.3±0.5#	7.5±0.6*#
VT	20.2±1.1†#	28.4±2.6* †#	8.8±0.8†#	12.7±0.9* †#
BD+VT	27.4±1.5† ‡#	39.0±1.4* † ‡#	12.9±1.4†#	18.3±1.8* † ‡#
CSD	−3.3±1.0†	−4.3±1.3†	−1.2±0.4†	−2.7±0.9†

Values are mean ± SE. n = 6 for each group.

*P<0.05 compared with saline.

†P<0.05 compared with INT.

‡P<0.05 compared with VT.

# P<0.05 compared with Sham.

## Discussion

The primary findings in the present study are that the CSAR is enhanced not only in CHF rats with bilateral vagotomy and arterial baroreceptor denervation, but also in intact CHF rats. The vagotomy and baroreceptor denervation augment the basal CSAR and the enhanced CSAR responses to Ang II in the PVN. The cardiac sympathetic denervation reversed the RSNA and MAP responses to capsaicin in both Sham and CHF rats. After cardiac sympathetic denervation, Ang II had no significantly effects on the CSAR in both Sham and CHF rats, but the Ang II-induced excitatory RSNA and MAP responses were reduced in CHF rats but not in Sham rats. These results indicate that the CSAR is also enhanced in intact CHF rats and the inputs of the arterial baroreceptor and vagal afferents inhibit the CSAR and the enhanced CSAR responses to Ang II in the PVN.

The cardiac sympathetic afferent stimulation increases sympathetic outflow and blood pressure in vagotomized and baroreceptor denervated dogs [30], rats [31] and cats [7]. It has been found that epicardial application of hydrogen peroxide (H_2_O_2_) in cervical vagotomized cat increases MAP, HR and LV dP/dt, while H_2_O_2_ in intact cat increases MAP but not HR and LV dP/dt. Furthermore, epicardial application of H_2_O_2_ in cats with T_1_–T_4_ ganglionectomy not only decreases MAP, HR and LV dP/dt, but also abolishes the excitatory effects of H_2_O_2_. These results suggest that activation of cardiac sympathetic afferents evokes cardiac excitatory and pressor responses, while activation of cardiac vagal afferents elicits inhibitory responses in normal cats [32]. Our previous studies have shown that the CSAR is enhanced in CHF rats [33] and renovascular hypertensive rats [28] and the enhanced CSAR partially contributes to sympathetic activation in these diseases. Ang II, AT_1_ receptors and superoxide anions in the PVN are involved in the enhanced CSAR in CHF rats [15], [17], [25] and hypertensive rats [13], [18], [29]. However, all these studies were carried out in vagotomized and baroreceptor denervated CHF or hypertensive rats. The present study was designed to determine whether the CSAR is enhanced in intact rats with CHF and whether the afferent activities of arterial baroreceptor and vagi inhibit the CSAR.

It is known that the cardiac afferent fibers travel with both cardiac sympathetic nerves and vagi [34]. The CSAR was enhanced in CHF rats with BD+VT, which is similar to our previous report [33]. Importantly, the CSAR was enhanced in intact CHF rats, suggesting that the enhanced CSAR also partially contribute to the sympathetic overdrive in intact CHF rats. The VT or BD+VT potentiated the RSNA and MAP responses to capsaicin in both Sham and CHF rats, suggesting that the afferent activities of arterial baroreceptor and vagi inhibit the CSAR. The CSD treatment reversed the RSNA and MAP responses to capsaicin in both Sham and CHF rats, suggesting that vagal afferent activities inhibit the sympathetic outflow and decrease blood pressure. Compared with Sham rats, the excitatory RSNA and MAP responses to capsaicin were enhanced in VT-treated CHF rats, but the inhibitory RSNA and MAP responses to capsaicin were not enhanced in CSD-treated CHF rats. These results indicate that the excitatory reflex mechanism mediated by sympathetic afferents prevails over the inhibitory mechanism mediated by vagal afferents.

AT_1_ receptor blockers (ARBs) and angiotensin-converting enzyme (ACE) inhibitors have been used to treat CHF [35], [36]. It has been shown that enhanced angiotensin in the PVN mediates the increased RSNA and AT_1_ receptors are up-regulated in the PVN in rats with CHF [37]. In CHF rats with BD+VD, microinjection of losartan, an antagonist of AT_1_ receptors, into the PVN abolished the CSAR and decreased the RSNA and MAP, while Ang II potentiated the CSAR and increased RSNA and MAP which is abolished by losartan [16]. In the present study, the increases in RSNA and MAP induced by the PVN microinjection of Ang II as well as the enhancement in the CSAR in intact CHF rats were greater than that in intact Sham rats. The VT or BD+VT significantly augmented the CSAR responses to Ang II but only induced a tendency in increasing RSNA and MAP responses to Ang II in both Sham and CHF rats. In both CSD-treated Sham and CHF rats, Ang II had no significantly effects on the CSAR and capsaicin still induced inhibitory RSNA and MAP responses. However, the Ang II-induced excitatory RSNA and MAP responses were reduced in CSD-treated CHF rats but not in CSD-treated Sham rats. These results suggest that the excitatory effects of Ang II in the PVN on the RSNA, MAP and CSAR are still greater in intact CHF rats than intact Sham rats, and that the baroreceptor and vagal afferent activities inhibit the effects of Ang II on the CSAR in both Sham and CHF rats. The role of Ang II in the PVN in increasing the RSNA and MAP partially attributes to its CSAR-enhancing effect. These results are supported by our recent findings that the firings of capsaicin-sensitive neurons in the PVN are greater in rats with BD+VT, VT or BD than intact rats after epicardial application of capsaicin, and that electrical stimulation of the vagal afferents inhibits the firings of capsaicin-sensitive neurons in the PVN in normal rats [38].

In conclusion, the CSAR is enhanced in intact CHF rats. The activities of the arterial baroreceptor and vagal afferents inhibit the CSAR and the enhanced CSAR responses to Ang II in the PVN.
